# The role of burnout prevention in promoting healthy aging: frameworks for the Semmelweis Study and Semmelweis-EUniWell Workplace Health Promotion Program

**DOI:** 10.1007/s11357-025-01780-w

**Published:** 2025-07-08

**Authors:** Virág Zábó, Andrea Lehoczki, Annamária Buda, Péter Varga, Monika Fekete, Vince Fazekas-Pongor, Marianna Moizs, Giorgia Giovannetti, Yura Loscalzo, Marco Giannini, Beatrix Busse, Miklos Kellermayer, Iveta Nagyova, Yongjie Yon, Róza Ádány, Béla Merkely, György Purebl, Zoltan Ungvari

**Affiliations:** 1 Institute of Preventive Medicine and Public Health, Semmelweis University, Budapest, Hungary; 2 Fodor Center for Prevention and Healthy Aging, Semmelweis University, Budapest, Hungary; 3 Institute of Behavioral Sciences, Faculty of Medicine, Semmelweis University, Budapest, Hungary; 4 Doctoral College, Health Sciences Division, Semmelweis University, Budapest, Hungary; 5 Department of Economics and Management, University of Florence, Florence, Italy; 6 Department of Health Sciences, School of Psychology, University of Florence, Florence, Italy; 7 Department of Linguistics, University of Cologne, Cologne, Germany; 8 Department of Biophysics and Radiation Biology, Semmelweis University, Budapest, Hungary; 9 Department of Social and Behavioural Medicine, Faculty of Medicine, PJ Safarik University in Kosice, Kosice, Slovakia; 10 WHO Regional Office for Europe, Copenhagen, Denmark; 11 Healthy Aging Section, European Public Health Association, Utrecht, The Netherlands; 12 Department of Public Health and Epidemiology, Faculty of Medicine, HUN-REN-UD Public Health Research Group, University of Debrecen, Debrecen, Hungary; 13 Heart and Vascular Center, Semmelweis University, Budapest, Hungary; 14 Vascular Cognitive Impairment, Neurodegeneration and Healthy Brain Aging Program, Department of Neurosurgery, University of Oklahoma Health Sciences Center, Oklahoma City, OK USA; 15 Stephenson Cancer Center, University of Oklahoma, Oklahoma City, OK USA; 16 Doctoral College, Health Sciences Division/Institute of Preventive Medicine and Public Health, International Training Program in Geroscience, Semmelweis University, Budapest, Hungary; 17 Oklahoma Center for Geroscience and Healthy Brain Aging, University of Oklahoma Health Sciences Center, Oklahoma City, OK USA; 18 Department of Health Promotion Sciences, College of Public Health, University of Oklahoma Health Sciences Center, Oklahoma City, OK USA

**Keywords:** Burnout, Ageing, Public health, Caring University Model Program, Employees, Health risk assessment, Occupational setting, Health promotion, Semmelweis Study, The Semmelweis-EUniWell Workplace Health Promotion Program

## Abstract

As the European Union undergoes a demographic shift toward an aging population, the rising prevalence of age-related diseases poses significant public health challenges. Across the EU, disparities in health indicators and life expectancies highlight the urgency of addressing factors that contribute to unhealthy aging. Burnout, characterized by emotional, physical, and mental exhaustion, has emerged as a critical determinant of healthy aging, with profound biological, psychological, and social implications. Burnout accelerates age-related decline, exacerbates health risks, and undermines overall well-being. This review explores the role of burnout in unhealthy aging, highlighting its underlying mechanisms and proposing actionable interventions. Workplace-based initiatives—such as the Caring University Model Program at Semmelweis University, which includes the Semmelweis Study and the Semmelweis-EUniWell Workplace Health Promotion Program developed within the European University for Well-Being (EUniWell) framework—offer a novel, integrated approach to embedding burnout prevention within broader public health strategies. By drawing on global best practices—including the Blue Zones model and Mediterranean lifestyle principles—and adapting them to diverse cultural contexts across Europe, these programs aim to foster resilience, reduce stress, and enhance workforce well-being. Addressing burnout as a critical determinant of health, these initiatives offer a scalable blueprint for promoting healthy aging and improving productivity throughout the EU. By incorporating burnout prevention into regional and national health policies, European countries can collectively extend healthspan, reduce healthcare costs, and enhance the quality of life for their aging populations.

## Introduction

Population aging represents a significant challenge for societies across the European Union (EU) and beyond^1^. Currently, over 100 million individuals aged 65 and older reside in the EU and UK, a figure projected to approach 150 million by 2050. This demographic shift, with the elderly comprising 21.2% of the EU population in 2022 and an anticipated 29.5% by 2050, highlights the growing burden of aging-related diseases [1]. Hungary faces particular challenges, including a rapidly aging and declining population, some of the EU's poorest health indicators—such as high rates of premature mortality from preventable and treatable causes, elevated cardiovascular disease burden, and one of the highest levels of avoidable hospitalizations—, and one of the shortest life expectancies in Europe [2].

Unhealthy aging, characterized by accelerated biological aging, early onset of chronic diseases, and reduced functional capacity, is a growing concern in Hungary [2] and many other member states of the EU [3]. Behavioral risks such as smoking, obesity, and low physical activity contribute to this trajectory, leading to high mortality and morbidity rates [2, 4]. Additionally, social determinants, including lower socio-economic status and unequal access to healthcare, exacerbate the burden of aging in the country. Addressing these challenges requires a multifaceted approach that goes beyond biomedical interventions to consider psychosocial assets, such as burnout, as critical factor which accelerates biological aging. Burnout—classified in the 11th Revision of the International Classification of Diseases (ICD-11) as an occupational phenomenon resulting from chronic workplace stress that has not been successfully managed—is characterized by three dimensions: emotional exhaustion, increased mental distance or cynicism toward one’s job, and reduced professional efficacy. While not a medical diagnosis, burnout is influenced by both external stressors and individual coping mechanisms, with dysfunctional coping strategies contributing to its onset and persistence [5, 6]. It has emerged as a critical factor influencing resilience and well-being, particularly in aging working populations. Evidence from “Blue Zones,” regions with exceptional longevity and health, underscores the importance of burnout prevention as a driver of healthier and more fulfilling lives [7–9]. In response to these challenges, Semmelweis University, the leading health sciences university in the region, has launched a dual initiative to combat unhealthy aging in Hungary [10, 11]. The Semmelweis Study [11], a longitudinal workplace cohort study involving all employees of the university, is designed to identify determinants of unhealthy aging, ranging from biological and psychological factors to lifestyle and environmental influences. Complementing this research arm, the Semmelweis-EUniWell Workplace Health Promotion Program aims to translate these findings into actionable interventions that improve the health and well-being of employees [12, 13].

Workplaces, where individuals spend much of their lives, offer a unique opportunity to implement health promotion strategies aimed at fostering healthier lifestyles and promoting healthy aging. The Semmelweis-EUniWell Workplace Health Promotion Model Program capitalizes on this potential by targeting key risk factors for unhealthy aging [12, 13]. Designed specifically for university employees, this program incorporates evidence-based interventions to promote physical, mental, and social well-being [12, 13]. As a cornerstone of Hungary’s National Healthy Aging Program, the initiative seeks to transform workplace environments to support healthier behaviors and foster resilience, creating a pathway toward a more sustainable approach to healthy aging.

Within the broader European context, Semmelweis University plays a pivotal role in the European University for Well-Being (EUniWell), a consortium committed to advancing societal well-being through collaboration, education, and research. This partnership connects Semmelweis with leading institutions such as the University of Birmingham (UK), Linnaeus University (Sweden), Nantes University (France), INALCO—The National Institute of Oriental Languages and Civilizations (France), the University of Cologne (Germany), the University of Florence (Italy), the University of Konstanz (Germany), the University of Murcia (Spain), the University of Santiago de Compostela (Spain), and Taras Shevchenko National University of Kyiv (Ukraine). Together, these universities work to exchange best practices and innovate solutions for improving health and well-being.

The Semmelweis-EUniWell Workplace Health Promotion Model Program emphasizes knowledge-sharing, collaboration, and the integration of diverse cultural perspectives to address the challenges of population aging [12, 13]. The program’s evidence-based framework supports the development of targeted interventions, cost analysis, and policy recommendations that can be adapted and scaled across the EU. By tackling the aging-related dimensions of demographic change, this initiative not only addresses immediate health challenges but also leverages the transformative potential of an aging population to foster inclusive societal well-being. Through its collaborative efforts, the program aspires to create innovative strategies that enhance healthy aging within university settings and beyond, contributing to the broader goals of improving public health across Europe.

Despite the growing recognition of the financial and social benefits of reducing work-related stress, a large-scale, longitudinal and multidisciplinary approach to healthy ageing of workers remains underexplored [14]. These initiatives represent a long-term commitment to understanding and addressing the drivers of unhealthy aging. By integrating epidemiological research with targeted health promotion efforts, the Semmelweis initiatives provide a unique platform for developing and testing strategies that can be scaled nationally and internationally. This paper delves into one particularly interesting factor—burnout—as a determinant of unhealthy aging and a target for workplace-based interventions [5, 6]. By examining the theoretical frameworks surrounding burnout, detailing approaches for its measurement and integration within the Semmelweis Study^11^, and identifying actionable workplace interventions, this manuscript offers insights into how burnout prevention can advance healthy aging. Additionally, it provides policy recommendations to ensure these interventions are scalable and effective across diverse settings. Drawing on global best practices and leveraging the strengths of the Semmelweis initiatives, this review seeks to promote a deeper understanding of how addressing burnout can foster resilience, well-being, and healthy aging across Europe.

## Learning from Blue Zones and the Mediterranean experience

Research on the Blue Zones—regions where people live exceptionally long and healthy lives—offers valuable insights into the prevention of burnout and the promotion of mental well-being across the lifespan. These regions, including Okinawa (Japan), Nauro Province in Sardinia (Italy), Nicoya (Costa Rica), Ikaria (Greece), and Loma Linda (California), share key psychosocial features such as a strong sense of purpose, social cohesion, and daily routines that promote balance and stress reduction—critical buffers against the chronic stress underlying burnout [7, 15–18]. More recently, so-called “Blue Zones 2.0” have emerged, exemplified by Singapore, where purposeful public policies have been used to shape environments that promote healthy aging, mental resilience, and social well-being. This demonstrates that the protective characteristics of Blue Zones are not limited to traditional or naturally occurring communities but can be actively cultivated through intentional design and governance.

A particularly powerful lesson from the Blue Zones is the centrality of purpose in life [13]. Known as *ikigai* in Okinawa [8, 9]and *plan de vida* in Nicoya^7^, this internal sense of meaning has been linked to reduced psychological distress and enhanced mental health. A well-defined purpose not only motivates individuals but also acts as a psychological anchor, reducing the risk of emotional exhaustion [19]. Equally important is the emphasis on balance and a slower pace of life. Inhabitants of Blue Zones prioritize rest, leisure, and meaningful social interactions over relentless productivity. This lifestyle rhythm helps counteract the performance-driven pressures that contribute to burnout in modern societies. Daily stress-reduction rituals—including prayer, meditation, time in nature, or short naps—are commonly practiced across Blue Zones [18]. These simple but consistent practices promote mental clarity, emotional regulation, and stress recovery, serving as effective tools for maintaining psychological well-being. Perhaps most notably, strong community bonds and multi-generational social support systems play a central role in mitigating feelings of isolation and fostering emotional resilience [20, 21]. Individuals remain connected through shared responsibilities and cultural rituals, underscoring the importance of belonging and mutual support in protecting against burnout.

Beyond the Blue Zones, the Mediterranean lifestyle offers a similarly compelling model for promoting mental health and preventing burnout [22–30]. In countries like Italy and Spain—home to two of Semmelweis University’s EUniWell partners—the integration of communal living, reflective practices, and social cohesion supports psychological resilience and emotional well-being. While the Mediterranean diet is well-known for its physical health benefits [31–34], its cultural context—marked by family meals, intergenerational care, and a culture of slowing down—also nurtures mental balance and a deep sense of connection [22, 23, 25, 26]. Importantly, dietary and lifestyle practices that support mental well-being are not exclusive to the Mediterranean region. Recent research from Finland, aligned with the Nordic Nutrition Recommendations (NNR), has also shown a significant association between adherence to a healthy diet and reduced burnout symptoms among municipal employees. In a large study by Penttinen et al. (2021), healthier dietary [29] patterns were linked to lower levels of emotional exhaustion, suggesting that nutritional quality may buffer against occupational stress and enhance mental resilience [35]. These cross-cultural insights—from Southern to Northern Europe—are highly relevant for Hungary’s healthy aging initiatives. They underscore the potential of integrating evidence-based dietary, cultural, and psychosocial practices to promote work-life balance, protect mental health, and prevent burnout in aging populations.

Building on these models, the Semmelweis-EUniWell initiatives aim to integrate such practices into workplace and community health strategies. The Semmelweis Study^11^ and the Semmelweis-EUniWell Workplace Health Promotion Program [12, 13], within the university’s “Caring University” framework [36], are uniquely positioned to adapt these global principles to Hungary’s cultural and institutional context. By promoting community-building in the workplace, creating opportunities for reflection and purpose-driven engagement, and aligning with Hungary’s traditions of resilience and family support, these programs can strengthen the psychosocial foundation for healthy aging. Through partnerships with Mediterranean institutions under the EUniWell umbrella, Semmelweis University can co-develop scalable, culturally adapted interventions that prioritize mental well-being and burnout prevention. In doing so, the university reinforces its leadership in innovative, evidence-based approaches to aging—recognizing that the path to a longer life must also support a more mentally resilient and emotionally fulfilling one.

## Burnout: concept and measurement

### Theoretical frameworks

Burnout is recognized as a work-related psychological syndrome comprising three core dimensions: emotional exhaustion, mental distancing or cynicism toward one’s job, and a decline in perceived professional efficacy [37]. The World Health Organization classifies burnout in the ICD-11 as an occupational phenomenon arising from unmanaged chronic workplace stress. Although not defined as a medical condition, burnout is shaped by both environmental stressors and individual psychological responses—particularly maladaptive coping strategies—which together influence its development and severity [5, 6]. Burnout is relevant both individually and organizationally, as it has been linked to physical and mental health issues (see for example [38, 39]) and is a predictor of work disability [40]. It manifests primarily as emotional fatigue, overload or exhaustion, depersonalization, poor view of ones self-competence and has traditionally been considered work-related mental-health impairment [41]. Burnout is secondarily manifested by physical or behavioral symptoms such as absenteeism, sick leave job turnover as well as physical health impediments [40, 42, 43]. As a key risk factor for psychological well-being throughout adulthood and old age, burnout is crucial for promoting mental, physical and social health. Several key theoretical frameworks provide insights into the role of burnout in human well-being and aging [37]. While diverse theoretical models and global practices provide a robust conceptual foundation, we have intentionally selected and adapted those elements most relevant to the Hungarian and European workplace context.

#### Maslach Burnout Model.^5^

It defines burnout as a syndrome comprising emotional exhaustion, depersonalization (cynicism), and reduced personal accomplishment which arises from chronic workplace stress, which drains emotional energy, fosters detachment from work and colleagues, and undermines a sense of achievement. These highlighted key dimensions provide insight into how workplace stress affects an individual’s mental health, job performance, and overall well-being.

#### Job demands-resources model

This model posits that burnout results from an [44, 45] imbalance between high job demands, such as workload and emotional strain, and insufficient job resources, including support and autonomy. This imbalance leads to the development of exhaustion and disengagement, which are recognized as the core components of burnout. The model underscores the critical need to manage job demands and strengthen resources as a strategy for effective burnout prevention.

#### Conservation of resources theory

It suggests that burnout arises when individuals experience or perceive a threat to their valuable personal, social, or material resources. This [46] stress initiates a downward spiral of resource depletion, ultimately culminating in burnout. The theory highlights the importance of protecting and replenishing resources as a critical strategy to mitigate burnout and its associated impacts.

#### Effort-reward imbalance model

This model posits that burnout occurs when [47] the effort invested in work is not sufficiently compensated by rewards, whether financial, emotional, or social. This imbalance generates significant stress, contributing to emotional exhaustion and diminished job satisfaction. The model emphasizes the critical role of equitable recognition and appropriate compensation in preserving employee well-being and preventing burnout.

These frameworks collectively underscore the multifaceted nature of burnout, integrating individual, organizational, and societal factors. Their application helps tailor interventions to address the specific drivers of burnout in various contexts.

### Mechanisms by which burnout contributes to unhealthy aging

#### Biological mechanisms

Burnout-induced chronic stress exerts profound effects on physiological systems, accelerating biological aging and increasing vulnerability to a wide range of age-related diseases. One of the most studied mechanisms is telomere shortening—the progressive erosion of protective chromosomal caps—which has been linked to impaired cellular repair, increased genomic instability, and earlier onset of cellular senescence [48, 49].

Another central mechanism involves dysregulation of the hypothalamic–pituitary–adrenal (HPA) axis. Prolonged activation of this system results in sustained hypercortisolism, impairing the body’s ability to regulate stress responses [50]. Chronic stress contributes to a persistent state of low-grade systemic inflammation [51], marked by increased pro-inflammatory cytokines. This inflammatory environment is a recognized driver of multiple age-related pathologies, including cardiovascular disease, osteoarthritis, and neurodegenerative disorders such as Alzheimer’s disease [51]. Inflammation also promotes oxidative stress [52], which further accelerates cellular aging by damaging DNA [53], lipids [54], and proteins [55], and impairing mitochondrial function[53]. These processes collectively disrupt metabolic homeostasis and increase the risk of conditions such as diabetes and hypertension, contributing to a downward spiral of physiological decline.

Beyond direct physiological mechanisms, burnout contributes to aging through behavioral and cognitive pathways [56]. Chronic job-related stress is likely associated with cognitive decline and an increased risk of dementia [57–59], potentially driven by the combined effects of neuroinflammation, sleep disturbances, and impaired brain plasticity. Burnout is also linked to poor health behaviors [60], including physical inactivity, unhealthy diet, and disrupted sleep patterns [61]. These behaviors further exacerbate metabolic dysfunction and reduce the body’s capacity for repair and resilience.

Together, these interconnected biological and behavioral mechanisms illustrate how burnout contributes to premature aging. The cumulative effect is a multifactorial burden that not only shortens lifespan but significantly diminishes healthspan—reducing quality of life and accelerating functional decline in older adults.

#### Psychosocial mechanisms

Psychosocial mechanisms play a critical role in the development and perpetuation of burnout, as interpersonal relationships and workplace environments shape stress levels, identity, and coping capacity [62]. Burnout frequently arises from stress inherent in asymmetrical professional relationships, where caregivers frequently prioritize others’ needs over their own [41]. This imbalance, coupled with high job demands, insufficient autonomy, and mismatched effort-reward dynamics, creates a fertile ground for burnout.

Key workplace factors contributing to burnout include excessive workload, mismatches between job demands and individual skills, insufficient autonomy, imbalances in effort and reward, poor work-life balance, limited control over career development, and exposure to interpersonal conflicts [45, 63–75]. Among healthcare professionals, additional stressors such as concerns about patient care, inadequate support, and frequent exposure to death and dying amplify burnout risk [76, 77]. Physicians may face challenges like perceived poor mental or physical health, low self-efficacy, and financial worries (including educational debt, costs associated with prolonged training periods and establishment of a practice and productivity-based reimbursement models, rising costs of malpractice insurance) [62, 78–81], while nurses often contend with heavy emotional labor and lack of autonomy [64–71]. Psychotherapists, particularly those who are younger or less experienced, face unique risks such as overinvolvement in client problems and emotional exhaustion [82]. Burnout is further exacerbated by societal pressures, such as cultural norms emphasizing constant achievement, the stigma surrounding mental health, and the expectation of emotional resilience in professional settings. In older workers, accumulated stress over the course of their careers intensifies these challenges, contributing to reduced emotional regulation, lower self-efficacy, and reliance on maladaptive coping strategies. Combined with reduced flexibility to change career paths later in life, the psychological effects of burnout—often overlapping with anxiety and depression—are particularly detrimental to aging populations [83]. Personality traits such as neuroticism [83], perfectionism [84], emotional dysregulation [85], reduced self-efficiency [86], and dysfunctional coping strategies [87]amplify vulnerability, while diminished social support and professional disengagement contribute to feelings of isolation. These dynamics underscore the critical need for targeted interventions that address both individual and systemic contributors to burnout.

A dynamic and multifaceted interaction exists among the biological and psychosocial processes of aging, the burden of age-related comorbidities, and the development or progression of burnout. Burnout profoundly influences aging by disrupting biological, psychological, and social pathways. Effective burnout prevention strategies, including stress management, resilience-building, and fostering social connections, can mitigate these harmful effects, promoting healthier aging trajectories. By addressing the root causes of burnout, interventions can enhance quality of life, extend healthspan, and foster resilience in aging populations. This underscores the transformative potential of integrating burnout prevention into healthy aging strategies, offering a comprehensive approach to improving well-being across the lifespan.

### Assessment of burnout

Accurately assessing burnout is essential for understanding its role in unhealthy aging and the potential for mitigation strategies. Several validated tools have been developed to measure burnout.

#### The Maslach Burnout Inventory (MBI)

Developed by Maslach and colleagues, the MBI is a widely used instrument that measures three core dimensions of burnout [88].

1. *Emotional Exhaustion* reflects the physical and emotional fatigue caused by prolonged stress.
2. *Depersonalization* assesses detachment and negativity in interpersonal interactions.
3. *Personal Accomplishment* gauges an individual’s sense of competence and efficacy.
4. The MBI’s multidimensional approach provides critical insights into the systemic and individual-level contributors to burnout, highlighting its impact on professional performance and psychological well-being.

#### The Burnout Measure (BM)

Pines and Aronson developed the BM to assess burnout as a multidimensional construct encompassing physical, emotional, and mental exhaustion [89]. This holistic framework emphasizes the interconnected nature of these dimensions, reflecting the pervasive and cumulative effects of chronic stress on individuals.

1. *Emotional Exhaustion* captures feelings of being overwhelmed and disengaged.
2. *Physical Exhaustion* highlights fatigue and stress-related symptoms.
3. *Mental Exhaustion* measures cognitive fatigue and diminished focus.

Together, these dimensions offer valuable insights into the multifaceted nature of burnout and its implications for psychological and physical well-being.

#### The Copenhagen Burnout Inventory (CBI)

The Copenhagen Burnout Inventory (CBI), developed by Kristensen his colleagues, represents a significant advancement in the measurement of burnout by prioritizing the subjective experience of exhaustion across multiple domains rather than focusing solely on role-specific factors [90]. Personal Burnout reflects overall physical and emotional exhaustion from prolonged stress, regardless of context. Work-Related Burnout focuses on fatigue caused by job tasks and demands, while Client-Related Burnout measures exhaustion from interactions with clients or patients. Together, these dimensions highlight the personal, professional, and interpersonal factors contributing to burnout.

Despite the availability of these tools, burnout remains underexplored in Hungary, particularly in workplace and aging-related contexts. A key strength of the Semmelweis Study is its focus on directly assessing burnout in the health sciences university-based cohort. The study uses the Maslach Burnout Inventory [88]to quantify burnout as a psychological endpoint. The Semmelweis Study aims to investigate the socio-economic, psychological, lifestyle, environmental, and biological determinants of metabolic, cardiovascular, neurocognitve, and psychological diseases, along with mortality. Burnout is a central psychological variable in this comprehensive analysis. In addition to quantitative assessments, incorporating qualitative approaches, such as open-ended questions or narrative interviews, can capture the subjective dimensions of burnout. Moreover, the study design allows for leveraging digital tools and surveys to enable longitudinal tracking of changes in burnout over time and its impact on health outcomes. By integrating these tools and approaches, the Semmelweis Study offers a unique opportunity to explore the role of work-related stress in shaping workplace health and aging trajectories. The findings have the potential to inform targeted interventions for burnout prevention and contribute to healthier aging outcomes.

## Burnout: a target for workplace-based stress reduction

Preventing and mitigating burnout is vital for promoting healthy aging, as it alleviates the physical, emotional, and cognitive strains that accumulate over time. Addressing burnout not only enhances individual well-being but also delivers societal benefits, including increased productivity and reduced healthcare costs.

### Mitigation of age-related chronic diseases

Workplace stress is a significant factor in the health of older adults. Prolonged stress, a key driver of burnout, is closely associated with chronic inflammation [91, 92], cardiovascular disease [91], and impaired immune function [92]. Preventing burnout helps regulate stress hormones [93]and production of inflammatory mediators [94–98], which reduces the risk of these conditions. Chronic stress has also been shown to contribute to cancer development, by promoting immunosuppression, inflammation, and tumor progression, further highlighting the systemic impact of unmanaged workplace stress [99]. By promoting physiological homeostasis, burnout interventions may play a crucial role in minimizing the burden of age-related chronic diseases [72, 73, 79–81, 100].

### Improved sleep quality

Burnout is strongly linked to poor sleep quality [61, 101–106], which exacerbates both physical and cognitive decline. Evidence-based strategies such as mindfulness and relaxation techniques have been shown to enhance sleep duration and quality [107, 108]. Adequate sleep supports cellular repair, healthy metabolism, clearance of toxic waste from the brain through the glymphatic system and thereby maintaining cognitive function [109–111], and overall vitality, providing a foundation for healthy aging [112–115].

### Maintenance of energy and activity levels

Burnout prevention preserves the energy needed for regular physical activity, a cornerstone of healthy aging [116]. Sustained engagement in exercise helps maintain muscle mass, bone density, and metabolic health while reducing the risk of frailty and age-related decline [116].

### Cognitive benefits

Cognitive health, a critical component of healthy ageing, is also positively affected by the prevention and absence of burnout [117]. Chronic stress linked to burnout has been shown to accelerate cognitive aging and increase the risk of neurodegenerative diseases, including Alzheimer’s [118]. Preventing burnout through stress-reduction strategies, including physical activity and meditation, protects brain health and reduces the risk of cognitive decline [119]. Practices that promote work-life balance and mindfulness foster neural resilience and cognitive flexibility [120], essential for maintaining mental sharpness throughout life.

### Mental health benefits

Burnout often overlaps with depression and anxiety [83]. Prevention strategies, such as fostering social connections [121]and improving emotional awareness and regulation [122], bolster psychological resilience. Effective burnout management encourages adaptive coping strategies and emotional stability [123], reducing the likelihood of mental health disorders and improving overall well-being in older adults [124, 125]. Workplace stress management strategies can also be adapted to other life domains, helping individuals build resilience and more effectively cope with diverse sources of stress.

### Social benefits

One of the most significant outcomes of burnout prevention is its impact on social well-being. Burnout frequently leads to withdrawal and strained interpersonal relationships [126]. By preventing burnout, individuals can invest time and energy into building meaningful social connections, which are critical for aging well. Prosocial behaviors in the workplace, such as mentorship and volunteering, foster a sense of connectedness and community. Moreover, reducing workplace stress enhances caregiving capacity [127], enabling older adults to balance caregiving responsibilities with personal well-being.

### Reduced mortality risk and enhanced quality of life

Burnout prevention and management strategies are strongly linked to improved health outcomes and reduced mortality risk [128]. Chronic stress—central to the experience of burnout—has been linked to a range of adverse health effects that cumulatively reduce life expectancy, including cardiovascular disease, metabolic syndrome, cancer and immune dysregulation [99, 129]. Multiple studies have shown that individuals experiencing high levels of work-related stress and burnout have a significantly increased risk of all-cause and cardiovascular mortality. For example, a large prospective cohort study in the Lancet found that job strain is associated with a 23% increased risk of coronary heart disease, independent of lifestyle factors such as smoking or physical inactivity [130]. Preventive strategies targeting burnout—such as improving work-life balance, promoting mental health literacy, enhancing social support, and fostering autonomy—have demonstrated efficacy in reducing stress levels and improving quality of life [131]. Interventions including mindfulness-based stress reduction, cognitive behavioral therapy, and workplace mental health programs have been shown to improve mood, and enhance subjective well-being [132].

Importantly, the benefits of reducing burnout extend beyond disease prevention. By preserving cognitive function, emotional resilience, and physical vitality, burnout prevention supports successful aging—a multidimensional concept encompassing independence, social engagement, and life satisfaction.

### Sustained productivity and independence

Finally, one of the most significant long-term benefits of workplace-based burnout prevention is its cumulative impact on sustained productivity and functional independence. By supporting both cognitive [133]and physical [119]functioning, burnout prevention enables older adults to remain engaged in work, daily tasks, and leisure activities for longer. This contributes not only to extended participation in the workforce but also to greater autonomy and quality of life during later life stages.

Workplace policies that actively promote stress reduction and well-being foster adaptability in the face of age-related changes. A resilient mindset—cultivated through supportive environments and effective stress management—helps individuals better navigate the physical, emotional, and social transitions that accompany aging. In doing so, burnout prevention contributes not just to individual well-being, but to the broader goal of healthy, active, and productive aging.

Positioning burnout as a modifiable risk factor for unhealthy aging has strategic implications. Burnout not only accelerates psychological decline but is also linked to biomarkers of biological aging, including chronic inflammation and oxidative stress. These mechanisms align with geroscience priorities and underscore why intervention in midlife work environments is essential. Acknowledging and intervening in burnout therefore supports both immediate mental health goals and long-term healthy aging trajectories.

## Semmelweis Study and the Semmelweis-EUniWell Workplace Health Promotion Model Program: integrating burnout prevention

Burnout has emerged as a critical public health challenge, with significant implications for individual well-being, workforce sustainability, and healthcare systems. In the United States alone, workplace stress is estimated to cost employers over $300 billion annually [134], highlighting the substantial economic impact of unmanaged occupational stress. The World Health Organization recognizes burnout as an occupational phenomenon resulting from chronic workplace stress that has not been successfully managed, and has called for systemic, preventive approaches to support mental health and well-being in the workplace [135]. WHO’s guidelines emphasize the responsibility of employers and governments to create supportive work environments that reduce psychological harm and promote resilience. In this context, the Semmelweis Study [11]—Hungary’s largest workplace cohort study—offers a unique opportunity to explore the determinants of burnout and implement multidimensional strategies for its prevention and mitigation.

Launched in 2024, the Semmelweis Study is the most comprehensive longitudinal workplace cohort study in Central Europe. It encompasses the entire workforce of Semmelweis University and aims to identify the key biological, psychological, social, and environmental determinants of healthy and unhealthy aging. The study integrates data from self-reported surveys, clinical examinations, physiological testing, and biomarker analyses. This transdisciplinary design enables the in-depth investigation of how workplace stress, including burnout, interacts with aging trajectories and health outcomes.

A central focus of the Semmelweis Study is the prevention and early identification of burnout among aging workers. The study collects extensive baseline and follow-up data across socio-economic status, lifestyle behaviors, mental health, and work environment, allowing for the identification of key burnout risk factors. Validated tools—including the Maslach Burnout Inventory (MBI) [88] and instruments assessing stress resilience—are used to measure burnout dimensions, coping strategies, and workplace stressors. These insights inform the development of targeted, evidence-based interventions tailored to individual and organizational needs. Preliminary data from the Semmelweis Study cohort reveal concerning trends: a significant percentage of participants report frequent feelings of emotional exhaustion and meet the threshold for moderate to severe burnout based on validated screening tools.

As part of the broader Semmelweis Caring University framework, the study is integrated with the Fodor Center for Prevention and Healthy Aging and the Semmelweis-EUniWell Workplace Health Promotion Model Program, which offers individualized lifestyle counseling and preventive health programs^10^. These services are adapted based on participants’ risk profiles and are designed to enhance psychological resilience and reduce burnout-related health risks. Workplace interventions informed by cohort data include flexible work arrangements, mental health promotion, and strategies to cultivate supportive and inclusive work environments—particularly for older employees.

A distinctive strength of the Semmelweis Study lies in its iterative and longitudinal design. Findings from each wave of data collection are used to refine subsequent survey instruments and intervention strategies, ensuring that approaches to burnout prevention remain responsive to emerging trends and contextual needs. The Study and the Semmelweis-EUniWell Workplace Health Promotion Model Program draw on frameworks such as the Mediterranean lifestyle, resilience training, and behavioral health science to encourage practices that support purpose, connectedness, and well-being—protective factors known to buffer against burnout. Regular follow-up assessments, planned every five years, provide robust longitudinal data to evaluate the effectiveness of implemented interventions. This enables real-time feedback for program refinement and supports the development of scalable models for workplace health promotion.

In addition to addressing psychosocial stressors, the Semmelweis Study investigates biological mechanisms linking burnout to unhealthy aging. By exploring pathways such as chronic inflammation and autonomic dysregulation, and immune function, the study seeks to identify novel biological markers of stress resilience and aging-related disease vulnerability. Furthermore, its culturally contextualized focus allows for the identification of Hungarian-specific workplace dynamics, values, and stressors, facilitating the development of culturally adapted prevention strategies.

Through this integrated, multidimensional approach, the Semmelweis Study and the Semmelweis-EUniWell Workplace Health Promotion Model Program aim to advance scientific understanding of burnout while informing its integration into national workplace health policies. Ultimately, these initiatives contribute to building healthier, more resilient, and productive aging trajectories—not only for Hungarian workers but as a model with relevance across Europe and beyond.

### Cultural adaptation of burnout prevention strategies

Effectively integrating burnout prevention strategies within the Hungarian context necessitates a nuanced understanding of the nation’s unique cultural, historical, and socio-economic background. Hungarian cultural norms emphasize perseverance and hard work, which, while fostering resilience, can inadvertently contribute to burnout when systemic challenges remain unaddressed.

Burnout among Hungarian professionals has become a pressing issue, particularly in high-stress sectors such as healthcare, education, and social services. Healthcare workers exhibit some of the highest burnout rates in the country, a situation exacerbated by the prolonged impacts of the COVID-19 pandemic [136]. A recent study [137] indicated that 64.4% of healthcare professionals experience mild to severe symptoms of burnout, which significantly correlate with elevated levels of depression and anxiety. These findings align with international data: a systematic review covering 76 studies worldwide concluded that the main risk factors for burnout during the pandemic included anxiety, depression, and insomnia, alongside sociodemographic factors such as being female, working as a nurse, or being in direct contact with COVID-19 patients [138]. These consistent patterns highlight the urgent need for targeted and context-sensitive interventions to strengthen the mental health and resilience of frontline professionals. In Hungary, chronic stress in the healthcare sector is further exacerbated by systemic challenges such as ongoing structural reforms, resource limitations, and the lack of systems-based approaches. Shifting dynamics in physician–patient relationships, declining social recognition of the profession, and the dominance of hospital-based care contribute to a strained working environment. Many professionals work simultaneously in the public and private sectors, leading to fragmented attention and burnout risk. Moreover, prevention and mental health promotion remain underprioritized in current health policy frameworks.

The burnout phenomenon is not limited to healthcare. Other demanding and emotionally taxing professions—such as mental health services, education, and lower-wage essential work—also face alarmingly high rates of burnout [139, 140]. Demographic vulnerabilities, including being single or lacking social support, further compound susceptibility to burnout and related mental health challenges [141]. Addressing these multifaceted risks requires comprehensive, cross-sectoral strategies that integrate mental health support into workplace structures, particularly for those working under high strain or in undervalued roles.

These findings underscore the need for culturally tailored interventions that address systemic challenges such as insufficient support systems, excessive workloads, and the pervasive stigma surrounding mental health. To effectively adapt burnout interventions to Hungary, the Semmelweis Study adopts culturally sensitive methodologies and integrates burnout assessment and prevention within its broader framework for workplace health. While tools like the Hungarian adaptation of the MBI [142] are already in use, further refinement is necessary to capture the unique manifestations of burnout in Hungarian society. Qualitative methods, including narrative interviews, could offer deeper insights into the specific risk factors and definitions of burnout shaped by Hungarian cultural values, whether derived from personal achievements, community contributions, or family dynamics.

Interventions promoting burnout prevention must align with national cultural norms and address inherent barriers. For example, workplace strategies emphasizing social connections, emotional well-being, and work-life balance could be seamlessly integrated into the Semmelweis-EUniWell Workplace Health Promotion Program. Additionally, incorporating culturally resonant practices, such as fostering intergenerational collaboration and community-building activities, could enhance the relevance and effectiveness of these initiatives. The “Family-friendly University” framework [36] at Semmelweis University provides a natural foundation for these efforts, emphasizing work-life balance, intergenerational cooperation, and the development of personalized coping strategies for work-related stress. By situating burnout prevention within this framework, the university not only addresses individual well-being but also promotes a supportive culture that extends to families and colleagues.

Ultimately, the cultural adaptation of burnout prevention strategies in Hungary—and more broadly across Central and Eastern Europe—requires a holistic and inclusive approach that ensures relevance across diverse demographic groups and professional contexts. By customizing assessments and interventions to reflect Hungary’s specific societal challenges and values, the Semmelweis initiatives can set a benchmark for addressing burnout within aging research and workplace health promotion across Central and Eastern Europe. This culturally sensitive framework holds the potential to amplify the efficacy of burnout mitigation efforts, fostering healthier, more resilient, and fulfilling lives for aging population.

These culturally responsive strategies are further strengthened through Semmelweis University’s active participation in the EUniWell alliance. Within this network, the Semmelweis-EUniWell Workplace Health Promotion Program serves not only as a national model, but also as a testbed for implementing and evaluating context-adapted interventions across diverse sociocultural settings. Collaborations with partner institutions enable the integration of region-specific insights into a shared framework for burnout prevention. This cooperative approach enhances the scientific and translational value of the Semmelweis Study, positioning it as a key contributor to a unified European strategy for addressing burnout as a determinant of unhealthy aging.

## Semmelweis-EUniWell Workplace Health Promotion Model Program: targeting burnout

The Semmelweis-EUniWell Workplace Health Promotion Program is a flagship initiative that integrates national and international efforts to promote healthy aging and prevent burnout in workplace settings. Embedded in the broader vision of the EUniWell alliance, the program aligns Semmelweis University’s institutional goals with a pan-European commitment to resilience, equity, and well-being. Drawing on insights from the Semmelweis Study and the shared expertise of EUniWell partners, the program designs and implements culturally relevant, evidence-based interventions that address workplace mental health and support healthy aging—particularly among aging professionals. By combining psychological resilience-building, organizational innovation, and community engagement, the program seeks to transform workplace environments into supportive ecosystems that reduce burnout risk and enhance overall well-being. Adapted to the Hungarian context but informed by best practices across Europe, the initiative aims to serve as a scalable model for workplace health promotion—one that is both scientifically rigorous and culturally sensitive.

### Workplace-based strategies for burnout prevention and healthy aging

Workplaces offer critical leverage points for promoting health, particularly in aging populations, where physical, mental, and social dimensions of well-being intersect. The Semmelweis-EUniWell Workplace Health Promotion Program adopts a comprehensive, multi-level strategy to prevent burnout, integrating organizational, managerial, and individual interventions to create resilient, inclusive, and health-promoting environments.

At the organizational level, interventions focus on restructuring the workplace to reduce chronic stressors and enhance flexibility. Key measures include the following: 1) Promoting work-life balance through flexible scheduling, remote work options, and limiting overtime; 2) Workload redesign to ensure equitable task distribution and prevent overburdening; 3) Establishing a culture of openness and psychological safety, encouraging regular breaks, vacations, and communication around mental health without stigma; 4) Offering access to Employee Assistance Programs (EAPs) and mental health resources.

At the managerial level, the program emphasizes leadership training to recognize early signs of burnout and actively support employee well-being. Key components include the following: (1) fostering empathetic leadership styles; (2) conducting regular check-ins and maintaining open, timely communication with staff; (3) strengthening team cohesion through conflict resolution and structured team-building activities; and (4) creating an enabling environment that supports positive change and psychological safety. These strategies align with the WHO's evidence-based approach to promoting mental health and preventing mental health conditions in the workplace [143].

While many interventions focus on individual stress management, the immediate workplace microenvironment plays a critical role in shaping burnout trajectories—particularly through social dynamics. In healthcare, there is a growing trend toward individualization at the expense of team cohesion, which undermines collegial support and collaborative resilience. Although technological advancement improves efficiency, it cannot replace the emotional and psychological value of human interaction in care delivery. Additional stressors include the negative portrayal of healthcare professionals in media communication, along with mounting administrative burdens that divert time and energy from meaningful patient care. These micro-level factors erode workplace morale, diminish emotional resilience, and compound the risk of burnout—especially in environments where professional identity and teamwork are essential. Therefore, the Semmelweis-EUniWell Workplace Health Promotion Program emphasizes the importance of restoring collaborative, human-centered work cultures. Rebuilding community in the workplace is essential, particularly in healthcare, where the emotional and relational dimensions of work are fundamental.

At the individual level, employees are empowered with tools for stress management and personal growth. These include 1) Mindfulness and relaxation techniques; 2) Resilience training and cognitive-behavioral strategies; 3) Physical wellness initiatives, such as fitness challenges or healthy eating programs; 4) Career development opportunities, skill-building workshops, and intergenerational mentorship programs, which promote knowledge transfer, purpose, and social support. 5) Innovative practices—such as a four-day workweek, compressed schedules, quiet zones, designated mental health days, return-to-work programs and anonymous feedback systems—complement these core strategies. These interventions collectively aim to promote productivity while preventing emotional exhaustion.

Case studies from leading organizations offer proof of concept. For example, Google incorporates mindfulness training and flexible work options to mitigate stress [144], while SAP’s global mindfulness program enhances focus and resilience [145]. IKEA prioritizes work-life balance by promoting flexible scheduling and limiting after-hours communication, demonstrating how organizational policies can reduce burnout [146].

The effectiveness of the Semmelweis-EUniWell program will be rigorously evaluated using a framework that tracks employee well-being, job satisfaction, and perceived burnout, as well as organizational indicators such as absenteeism, retention, and productivity. To assess burnout and its trajectory over time, the Semmelweis Study employs a comprehensive, multi-modal assessment strategy. Burnout symptoms are measured using validated instruments such as the Maslach Burnout Inventory (MBI), which captures key dimensions of exhaustion, cynicism, and professional efficacy. In addition to self-reported metrics, the program monitors absenteeism, turnover rates, and job satisfaction through institutional data systems. Health outcomes, including stress-related illnesses and mental health improvements, will also be tracked. Mental health status (e.g., anxiety, depression, and sleep quality) is assessed using validated questionnaires integrated into the Semmelweis Study. By addressing both psychosocial needs and structural drivers of burnout, the program not only aligns with the EUniWell mission of building resilient communities but also sets a precedent for scalable, evidence-based workplace health promotion across Central and Eastern Europe.

### Developing a Mediterranean-inspired framework for healthy aging

To strengthen its cultural resonance and broaden its appeal, the program draws on the Mediterranean lifestyle, incorporating principles of balance, community, and purpose that align closely with Hungarian traditions. While the Mediterranean diet is renowned for its cardiovascular and metabolic benefits [31–34], its value also lies in the accompanying social practices—such as communal meals, intergenerational bonds, and time for rest and reflection—that offer powerful tools for combating burnout. While the Mediterranean model offers guiding principles for community, nutrition, and balance, the Hungarian context brings its own cultural strengths—such as strong intergenerational ties, culinary traditions, and shared family rituals. Yet these traditions have weakened under the pressures of modern life, with the rise of processed food consumption, erosion of extended family networks, and limited time for leisure. The Semmelweis program seeks to revitalize these Hungarian strengths by merging them with Mediterranean principles to foster a hybrid approach tailored to the realities of Hungarian workplaces.

By integrating these elements, the program can create culturally attuned interventions that are both relevant and sustainable. Operationalizing this framework involves shared meals to promote healthy eating while strengthening social cohesion and structured leisure and reflection activities, including mindfulness practices and downtime, to enhance emotional resilience and reduce chronic stress. By synthesizing Hungarian and Mediterranean strengths, this hybrid model supports holistic well-being—addressing not just nutrition and exercise, but also connection, meaning, and emotional balance. It reimagines workplace health promotion as a culturally rich, socially embedded strategy for aging well.

Looking ahead, this Mediterranean-Hungarian framework offers a blueprint for scalable, cross-cultural intervention. Through EUniWell collaborations and pilot programs, Semmelweis University can further refine and disseminate this model, contributing to a European standard for culturally sensitive burnout prevention and workplace well-being. In doing so, it positions Hungary at the forefront of innovative, inclusive strategies for promoting resilience and longevity in the aging workforce.

While individual-focused interventions are necessary, they are not sufficient. Strengthening the immediate community—particularly the social microenvironment at work—is critical to sustaining positive change. The success of intervention programs depends not only on personal coping skills but also on the presence of socially cohesive teams. Within Hungary’s healthcare system, fostering a renewed sense of workplace community represents an untapped source of resilience. Semmelweis University's experience in cultivating workplace solidarity offers a strong foundation for embedding this community-level approach into national burnout prevention strategies.

## Burnout prevention: an essential component of national healthy aging strategies

As Hungary—and much of Europe—faces the challenges of an aging population, addressing psychosocial determinants of health has become an urgent priority [3, 147]. Burnout, increasingly recognized as a critical factor in unhealthy aging, has far-reaching implications not only for individual health but also for the sustainability of healthcare systems and national productivity. The integration of burnout assessment and intervention into the Semmelweis Study and the Semmelweis-EUniWell Workplace Health Promotion Program represents a transformative step toward establishing a comprehensive National Healthy Aging strategy.

Burnout contributes to accelerated biological aging, increased chronic disease burden [51–55], and diminished psychological well-being [148]. Effective burnout prevention enhances resilience, supports cognitive and emotional health, and fosters health-promoting behaviors—such as physical activity, social engagement, and adherence to medical care. These outcomes, in turn, reduce healthcare costs and extend the period of life spent in good health (healthspan). By embedding burnout prevention into national public health policy, Hungary can amplify the impact of its healthy aging initiatives. The Semmelweis model offers an evidence-based framework for workplace-based interventions that are adaptable and scalable. National campaigns grounded in this framework could promote awareness of burnout’s risks and protective strategies, with messaging tailored to different demographics. Such campaigns would not only shift public attitudes but also normalize the pursuit of emotional well-being and psychological resilience as essential components of healthy aging.

The effective prevention of burnout—and its integration into national healthy aging strategies—requires more than awareness; it demands structured, systemic implementation in everyday environments. Among these, the workplace represents the most strategic and scalable setting for intervention. Workplaces concentrate large segments of the adult population, offer continuous access to individuals throughout their working lives, and provide natural platforms for delivering mental health promotion, prevention, and support.

The Semmelweis-EUniWell Workplace Health Promotion Program offers a replicable and evidence-based model for national implementation. Two complementary pathways are proposed to bring this model to scale across Hungary.

1) Integration through the national network of Health Promotion Offices (HPOs):

The structured rollout of the Semmelweis Workplace Health Promotion Program through Hungary’s existing HPO network would allow coordinated, institution-based interventions to be implemented in both public and private sector organizations. HPOs could serve as hubs for delivering training on burnout prevention, facilitating employee screening and support programs, and embedding well-being into organizational culture. These interventions could include stress management workshops, team-based resilience-building, promotion of work-life balance, and early recognition of burnout symptoms.

2) Deployment through the Semmelweis Integrated Primary Care Team Model as part of Primary Care Reform:

Burnout prevention must also be available beyond formal employment settings. The Semmelweis Integrated Primary Care Team Model is a comprehensive initiative developed by Semmelweis University to enhance primary healthcare services in Hungary [10]. This model brings together a multidisciplinary team—including family physicians, preventive medicine specialists, public health professionals, dietitians, physical therapists, and mental health professionals—to provide coordinated and holistic public health services and patient care. The program aims to improve health outcomes by integrating preventive services and promoting healthy aging within the community. It serves as a pilot project for national health promotion and disease prevention programs, demonstrating the effectiveness of collaborative, team-based approaches in primary care settings [10]. By fostering close collaboration among various healthcare professionals, the Semmelweis model enhances the delivery of preventive services and supports the ongoing reform of primary healthcare in Hungary.

The Semmelweis Integrated Primary Care Team Model enables primary care teams to deliver community-based mental health services. In this framework, family physicians are trained to recognize early signs of burnout and stress-related disorders, and refer individuals for timely psychosocial intervention. Mental health professionals embedded within primary care practices can then offer counseling, group-based therapy, or digital mental health tools tailored to the needs of aging individuals. This not only increases access to care but embeds mental health support into the most accessible level of the healthcare system.

Together, these two pathways allow for a dual approach: targeted prevention in workplaces, and accessible treatment in community settings. Workplace health promotion programs can incorporate preventive elements—such as psychological safety training, leadership engagement, and resilience education—while primary care can serve as a safety net for individuals already experiencing burnout-related symptoms.

By leveraging these implementation channels, Hungary has the opportunity to establish a nationally coordinated, community-rooted, and workplace-anchored burnout prevention framework. This approach strengthens resilience across the population, reduces the burden of mental health disorders, and positions burnout prevention as a central and practical element of healthy aging policy.

### Policy recommendations for national healthy aging programs

To translate this vision into actionable policy, the following strategic recommendations are proposed:

- 1. Integrate burnout prevention into national health plans:
- Recognize burnout as a major psychosocial determinant of health and embed its prevention and management into national frameworks for healthy aging and mental health.
- 2. Expand access to mental health services:
- Invest in early identification, counseling, and treatment for stress-related conditions. Ensure equitable access across urban and rural areas, and among high-risk occupational groups such as healthcare and social workers.
- 3. Support employer-led health initiatives:
- Encourage organizations to implement workplace wellness programs modeled on the Semmelweis-EUniWell framework, with incentives such as tax relief, accreditation, or public recognition. These programs should emphasize work-life balance, psychological safety, and resilience training.
- 4. Launch targeted public awareness campaigns:
- Educate the population on the risks of burnout and promote accessible, evidence-based strategies for prevention—including stress management, healthy relationships, and community engagement. Campaigns should use diverse media formats and be adapted for various population segments.
- 5. Foster research and innovation:
- Allocate funding to multidisciplinary research on burnout, including its biological and psychosocial mechanisms, long-term health impacts, and effective intervention strategies. National studies should build on the Semmelweis Study to guide policy and program development.
- 6. Monitor and evaluate impact:
- Develop indicators to assess the effectiveness of burnout prevention strategies in improving population health outcomes, workplace productivity, and quality of life among older adults. Use data to iteratively refine programs.

By implementing these policy measures, Hungary can take a leading role in embedding burnout prevention into its national healthy aging agenda. These actions will not only mitigate the burden of chronic stress but also empower individuals to lead longer, healthier, and more fulfilling lives—benefiting society at large.

## Conclusion

Burnout is increasingly recognized as a critical determinant of unhealthy aging, with profound implications for individual well-being, workforce sustainability, and public health systems. As Hungary and other European nations grapple with the challenges of aging populations, the need for integrated, systemic solutions has never been greater.

Through the Semmelweis Study and the Semmelweis-EUniWell Workplace Health Promotion Program, we are developing a pioneering model for embedding burnout prevention into national aging strategies. These initiatives combine rigorous epidemiological research, culturally adapted interventions, and international collaboration to identify and address the psychosocial factors that drive accelerated aging. By positioning the workplace as both a site for data collection and an arena for implementation, this dual approach offers a scalable and evidence-based framework for reducing stress, enhancing resilience, and extending health span.

The integration of the Semmelweis model into primary care—through the Semmelweis Integrated Primary Care Team Model—and the national Health Promotion Office network provides a unique infrastructure for delivering mental health and burnout prevention services to broad segments of the population. This dual-channel implementation strategy ensures that interventions are accessible, context-sensitive, and sustainable.

Burnout prevention is not only a means of reducing disease burden and healthcare costs; it is also a pathway to more engaged, purposeful, and fulfilling lives across the life course. Hungary’s commitment to this vision, supported by international partnerships and informed by global best practices, has the potential to set a new standard for public health policy in Europe. By prioritizing mental well-being and workplace health promotion as key pillars of national aging strategies, Hungary is taking bold steps toward a future in which people not only live longer—but thrive as they age.

## Data Availability

N/A.
